# Esophageal tissue immunoglobulin G4 in eosinophilic esophagitis and its correlation with serum-specific IgG4 to six foods

**DOI:** 10.3389/falgy.2026.1750690

**Published:** 2026-01-26

**Authors:** Eva Macías, Ana Menéndez-Ramos, Antonio Velasco-Guardado, Jose A. Muñoz-León, Marta Rodríguez-González, Ignacio Dávila

**Affiliations:** 1 Allergy Department, University Hospital of Salamanca, Instituto de Investigación Biomédica de Salamanca (IBSAL), Salamanca, Spain; 2 Digestive Department, University Hospital of Salamanca, Instituto de Investigación Biomédica de Salamanca (IBSAL), Salamanca, Spain; 3 Pathological Anatomy Department, University Hospital of Salamanca, Salamanca, Spain

**Keywords:** eosinophilic esophagitis, food allergy, IgG4, immunoglobulin, plasma cell

## Abstract

**Background & aims:**

Eosinophilic esophagitis (EoE) is a chronic inflammatory disorder in which IgG4 involvement is unclear. We aimed to evaluate immunoglobulin G4 (IgG4) in eosinophilic esophagitis (EoE) and investigate the correlation among IgG4-positive plasma cells in esophageal tissue, total serum immunoglobulin E (IgE), specific IgE, and specific IgG4 (sIgG4) levels to six foods (milk, egg, wheat, soy, peanut, and seafood).

**Methods:**

A retrospective observational study with prospective patient inclusion was conducted from 2017 to 2024 in a real-world setting. Clinical, endoscopic, therapeutic, and outcome characteristics of the patients were collected. Positive staining was defined as >10 IgG4-positive plasma cells per HPF. Peripheral blood analyses included measurements of total IgE, specific IgE, and sIgG4.

**Results:**

Seventy-eight patients were included. Patients with positive histological staining exhibited a significantly higher proportion of endoscopic edema (16.1% vs. 2.1%) (*p* = 0.045; RR = 8.8, 95% CI:1–79.8) and a greater need for second-line treatment (64.5% vs. 41.3%) (*p* = 0.04; RR = 2.5, 95% CI:1–6.6). Patients with positive IgG4 histological staining exhibited significantly higher median concentrations of serum sIgG4 to milk casein (50.1 mg_A_/L vs. 11.2 mg_A_/L; *p* = 0.005; r = 0.31) and egg (63.4 mg_A_/L vs. 20.1 mg_A_/L; *p* = 0.011; r = 0.28) than those with negative stain. Patients who did not show histological response to first-line treatment had significantly higher concentrations of sIgG4 to casein (31.8 mg_A_/L vs. 18.6 mg_A_/L, *p* = 0.03; r = 0.26) and egg (45.46 mg_A_/L vs. 19.2 mg_A_/L, *p* = 0.03; r = 0.27).

**Conclusion:**

Serum sIgG4 levels to casein and egg were associated with infiltration of IgG4-positive plasma cells in esophageal tissue and a worse disease prognosis.

## Graphical Abstract

**Figure:**
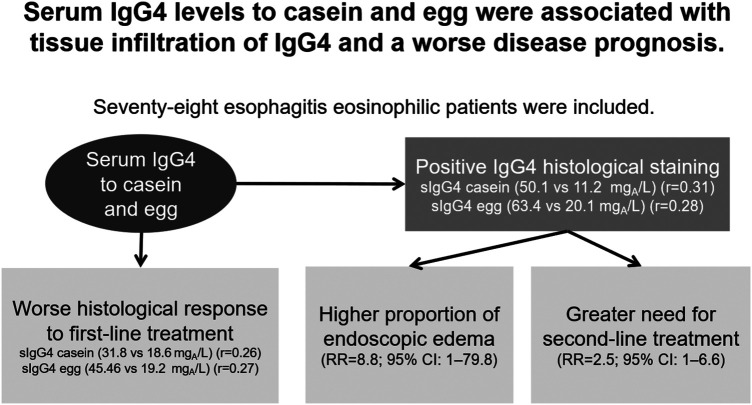

## Highlights Box

What is already known about this topic?

Eosinophilic esophagitis (EoE) is a chronic inflammatory condition characterized by eosinophil infiltration in the esophagus, often triggered by food antigens.
The role of IgG4 in EoE pathogenesis is unclear, with prior studies suggesting its presence in esophageal tissue and potential association with food-specific immune responses, but data on its clinical utility remain limited.

Significant and/or new findings of this study

In a cohort of 78 EoE patients, 39.7% showed positive esophageal IgG4 staining (>10 IgG4-positive plasma cells/HPF), which was associated with increased endoscopic edema (RR = 8.8, 95% CI: 1–79.8) and a greater need for second-line treatment (RR = 2.5, 95% CI: 1–6.6).
Patients with positive IgG4 staining had significantly higher serum-specific IgG4 levels to milk casein (50.1 vs. 11.2 mgA/L, *p* = 0.005) and egg (63.4 vs. 20.1 mgA/L, *p* = 0.011), indicating a correlation with tissue infiltration.
Non-responders to first-line treatment exhibited elevated serum IgG4 levels to casein and egg, suggesting that these markers may predict a worse disease prognosis and guide personalized dietary interventions.

## Introduction

Eosinophilic esophagitis (EoE) is a chronic type 2 (T2) inflammatory condition characterized by the isolated and abnormal infiltration of eosinophils in the esophagus, presenting a spectrum of symptoms that include dysphagia, slow eating, vomiting, or food impaction (1). Its incidence and prevalence have significantly increased over the past decade (2). Early diagnosis through histological analysis, combined with appropriate management strategies, often involving dietary modifications and/or medications, is essential to alleviate symptoms and prevent long-term complications (3). Nowadays, the diagnosis, monitoring, and evaluation of treatments require repeated esophageal biopsies, which involve multiple esophagogastroduodenoscopies (EGD). Thus, finding less invasive methods to diagnose and monitor EoE is crucial. Numerous efforts have been made to identify a distinctive factor for predicting EoE, with a primary focus on peripheral blood. However, no specific predictive marker has been found. Although the underlying pathophysiology is incompletely understood, the current hypothesis is that food antigens are presented to the gastrointestinal tract, triggering T cells to produce a cascade of Th2 inflammatory cytokines, resulting in Th2 lymphocyte–predominant inflammation (4). Presently, data do not demonstrate a significant role for IgE in the pathophysiology of EoE (4–6).

A potential role of IgG4 has been suggested in EoE. Recent data suggested that the cytokine profile in the esophageal mucosa microenvironment promoted the production of IgG4 in EoE (4). Thus, regulatory T-cells can produce IL-10, which enhance IgG4 production in chronic exposure settings (5, 7–9). Notwithstanding, its precise role remains unknown, and studies have yielded contradictory results (10, 11). Previous studies have suggested that the presence of IgG4 in the esophagus of subjects with EoE indicates chronic exposure to an antigen in atopic individuals, which appears to be associated with a specific IgG4 response, and that such an antigen could contribute to the pathogenesis of EoE (8).

Given the current lack of knowledge, we aimed to evaluate IgG4 in EoE. The specific goals were to study whether there were differences in IgG4 levels between patients diagnosed with EoE with or without concomitant food allergies and to establish whether there is a correlation between esophageal stain for IgG4, total IgE, specific IgE, and specific IgG4 levels to the six foods most frequently associated with EoE (milk, egg, wheat, peanut, soy, fish, and seafood).

## Methods

### Study Design

A retrospective observational study with prospective patient inclusion was conducted from 2017 to 2024 in a real-world setting. The study population consisted of patients with a clinical and histological diagnosis of EoE. The study was multidisciplinary, involving allergists, gastroenterologists, and pathologists.

The study design is hybrid: patient inclusion and sample collection (esophageal biopsies and blood) were conducted prospectively over 7 years, as patients were consecutively enrolled upon EoE diagnosis during routine clinical care. The retrospective component involved the subsequent analysis of stored samples and clinical data, which were reviewed and processed after the recruitment period to evaluate histological and serological findings.

The study was approved by the Clinical Research Ethics Committee (CEIm code: PI2020/10/592) of the University Hospital of Salamanca. All patients provided written informed consent prior to their inclusion in the study.

### Methodology

Inclusion criteria: Patients over 18 years of age with a confirmed diagnosis of EoE (compatible esophageal biopsy) were invited to participate in the study. Upon informed consent, patients were referred to the Allergy department for the allergy study and the collection of serum samples. Once enrolled in the study, patients underwent standard follow-up and treatment for EoE, managed collaboratively by the Allergy and Gastroenterology Departments.

### Endoscopic procedure and histological analysis

Endoscopic examinations were performed using an Olympus® GIF-H190 high-definition gastroscope with an Olympus® EVIS EXERA III CV-190 processor (Olympus Corporation, Tokyo, Japan). Endoscopic features were assessed using the Eosinophilic Esophagitis Endoscopic Reference Score (EREFS), a validated system that grades edema, rings, exudates, furrows, and strictures (12).

Conventional biopsies were obtained from the proximal and distal esophagus and placed in separate containers (with at least three biopsies per location) based on the European guidelines for EoE of the United European Gastroenterology (12). Samples were collected prospectively at the time of diagnosis: esophageal biopsies were fixed in formalin, embedded in paraffin, and stored at room temperature per standard pathology protocols. Histological analysis was conducted using standard hematoxylin-eosin staining, with routine pathological interpretation of the samples, including eosinophil counts per high-power field (HPF). Subsequently, immunohistochemical staining was performed using the Leica® Bond III kit with a mouse monoclonal antibody against human IgG4 (clone MRQ-44, Master Diagnóstica®, Granada, Spain). IgG4 staining in the interstitial space was assessed. The number of IgG4-positive plasma cells per HPF and the total number of plasma cells in the sample were also quantified. Positive staining was defined as more than 10 IgG4-positive plasma cells per HPF, regardless of interstitial staining, based on histological diagnostic criteria used in other IgG4-related diseases (13, 14). In IgG4-related diseases, histological positivity is defined by consensus criteria that combine morphological features and immunohistochemical quantification. Key findings include a dense lymphoplasmacytic infiltrate, storiform fibrosis, and obliterative phlebitis, with emphasis on IgG4 staining in plasma cells. There is no specific cutoff for “interstitial staining” *per se*, as this refers to the pattern of lymphoplasmacytic infiltrate and fibrosis in the tissue interstitium, which is qualitative and must be present to support the diagnosis, but it is not quantified. The number of IgG4-positive plasma cells per high-power field (HPF; typically 400×) is an essential quantitative criterion, with cutoff values varying by affected organ and sample type. In general, >10 IgG4+/HPF is a minimum threshold, but must be combined with other histological features.

### Allergy study

Skin prick tests for food and aeroallergens were performed according to the standardized procedures outlined in the 2020 European Academy of Allergy and Clinical Immunology (EAACI) position paper on skin tests in allergic diseases (15).

Blood samples were processed for serum and stored at −80 °C in secure freezers with temperature monitoring and backup systems to ensure sample integrity. Quality controls included visual inspection of biopsy slides for adequacy, verification of freezer logs for temperature stability, and exclusion of any degraded samples (*n* = 2 excluded due to insufficient material). Peripheral blood analyses included measurements of total IgE, specific IgE, and specific IgG4 using the ImmunoCAP 250 system (Thermo Scientific®, Phadia Spain S.L., Barcelona, Spain) to detect proteins from milk, egg, wheat, peanut, soy, fish, and seafood.

ImmunoCAP Specific IgG4 is an immunoassay for the quantitative determination of allergen-specific IgG4 antibodies in human serum or plasma. ImmunoCAP Specific IgG4 should be used in clinical laboratories with the Phadia 200, 250, 1000, 2,500, or 5000N instruments. In our study, the Phadia 250 was used. For ImmunoCAP Specific IgG4, results are expressed as concentration in mgA/L, where A represents allergen-specific IgG4 antibodies. The limit of quantification is 3 µgA/L, corresponding to 0.3 mgA/L in an undiluted sample (CLSI. Evaluation of Detection Capability for Clinical Laboratory Measurement Procedures; Approved Guideline—Second Edition. CLSI document EP17-A2. Wayne, PA: Clinical and Laboratory Standards Institute; 2012).

The extracts used were the same for IgG4 as for IgE and were chosen because they are the food allergens most frequently associated with eosinophilic esophagitis: milk, egg, wheat, peanut, soy, fish, and seafood. These have been studied in numerous published articles on eosinophilic esophagitis.

Regarding assay variability, the accuracy of the measured values of ImmunoCAP Specific IgG4 is, with a 95% probability, within ± 7% of the WHO IgG4 reference value, and the bias of ImmunoCAP Specific IgG4 Calibrators is, with a 95% probability, ±3% compared to the WHO IgG4 reference value.

Positive thresholds were defined as total IgE levels greater than 120 kU/L, specific IgE levels greater than 0.35 kU_A_/L, and specific IgG4 levels greater than 10 mg_A_/L. For milk-specific IgG4, analyses for casein and β-lactoglobulin were performed, as the manufacturer advises against using the milk ImmunoCAP Specific IgG4 assay due to native IgG content or nonspecific background binding, which may lead to misleading results (16). That does not apply to casein or beta-lactoglobulin, which are major milk allergens and were used for that reason. In recent studies, casein has also been used instead of whole milk extract and represents a validated method (17).

### Statistical analysis

Data were analyzed using IBM® SPSS® Statistics for Windows, Version 28.0 (IBM® Corp., Armonk, NY: 2021). Descriptive statistics, Student's *t*-test, Chi-square test, Fisher's exact test, analysis of variance (ANOVA), Pearson and Spearman correlation analyses, and multivariate multiple regression analyses were performed. Normality was assessed using the Kolmogorov–Smirnov test for quantitative variables. All variables showing non-normal distributions were analyzed using nonparametric tests (Mann–Whitney *U*-test) for comparisons.

For multivariate analysis, binary logistic regression was used, including variables with *p*-values < 0.1. Statistical significance was considered at *p* < 0.05.

## Results

### Baseline patient characteristics

Seventy-eight patients with a histopathological diagnosis of EoE (>15 eosinophils per HPF in biopsy) were included with a median follow-up of 27 months (interquartile range, IQR: 12.7–36). The median age of the patients was 35.5 years (IQR: 27.7–45.5).

The clinical, endoscopic, therapeutic, and outcome characteristics of the patients are summarized in Table 1.

**Table 1:** Clinical, endoscopic, therapeutic, and outcome characteristics of the patients.

Characteristics	Definition	*N*	%
Clinical features			
Gender (male)		58	74.4
Family history of atopy		30	39
Atopic dermatitis		13	16.7
Rhinoconjunctivitis		60	76.9
Allergic asthma		27	34.6
Food allergy		34	43.6
Symptoms			
	Dysphagia	64	82.1
	Food impaction	36	46.2
	Heartburn	20	25.6
	Chest pain	3	3.8
Endoscopic findings (EREFS features)			
	No findings	6	7.7
	Edema	6	7.7
	Rings	44	56.4
	Cotton-like exudates	34	43.6
	Linear furrows	66	84.6
	Stenosis	4	5.1
Initial therapy			
	Proton Pump Inhibitors (PPI)	66	84.6
	Swallowed corticosteroids	5	6.4
	Empirical diet	3	3.8
	Targeted diet	3	3.8
	No treatment	1	1.3
Need for second-line treatment		38	65.4
	Swallowed corticosteroids	23	29.5
	PPI	6	7.7
	Empirical diet	2	2.6
	Elemental diet	2	2.6
	Targeted diet	5	6.4
Pneumatic dilation		3	3.8
Response to PPI		39	54.2
Histological response (<15 eos/HPF) to first-line therapy		20	25.6

### Histological findings in esophageal biopsies

In the histological analysis, all patients showed >15 eosinophils per HPF (inclusion criteria). The median eosinophil count was 86 per HPF (IQR: 47–131) and 188 per mm² (IQR: 118–300).

Sample adequacy based on basal lamina characteristics was minimal (only axes) in 55.1% (*n* = 43), scarce (<10%) in 26.9% (*n* = 21), and adequate (>10%) in 15.4% (*n* = 12). Eosinophil degranulation was observed in 89.7% (*n* = 70). Basal lamina hyperplasia was identified in 71.8% (*n* = 56) of the samples. Eosinophil abscesses were present in 50% of patients (*n* = 39).

**Figure 1:**
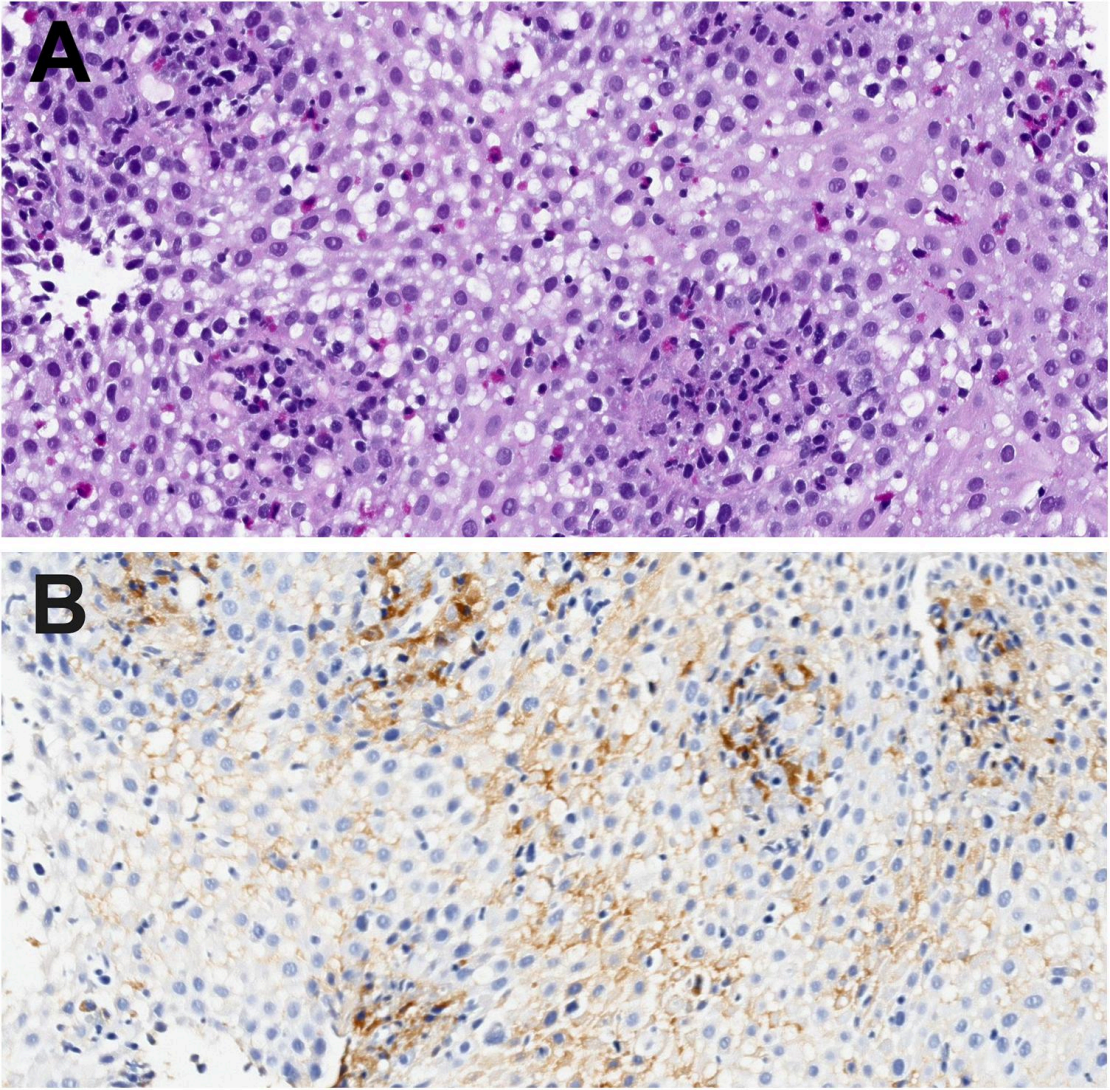
**(A)** hematoxylin and eosin stain (400× magnification). Esophageal epithelium meeting pathological criteria for eosinophilic esophagitis. >15 eosinophils per high-power field. Also observed are eosinophil degranulation and spongiosis of the squamous epithelium. **(B)** IgG4 stain (400× magnification). Intense intracytoplasmic granular and reticular positivity in the esophageal epithelium. Also note abundant plasma cells in the lamina propria with positive cytoplasmic staining.

Regarding IgG4 staining, both interstitial and plasma cell staining were identified (Figure 1). Interstitial IgG4 staining was observed in 23.1% (*n* = 18) of patients.

The median total plasma cell count for the samples was 80 (IQR: 30–180). At least one IgG4-positive plasma cell per HPF (IgG4+/HPF) was observed in 64.5% of patients (*n* = 49). The median IgG4+/HPF plasma cell count was 5 (IQR: 0–23).

More than 10 IgG4+/HPF plasma cells were identified in 39.7% (*n* = 31) of patients. Among patients with >10 IgG4+/HPF plasma cells, 51.6% exhibited positive interstitial IgG4 staining compared to 6.4% of those with <10 IgG4+/HPF plasma cells (*p* < 0.001, RR = 4.7; 95% CI: 1.6–13.4) (Table 2).

**Table 2:** Summary of histological findings in esophageal biopsies from patients with eosinophilic esophagitis (EoE).

Histological finding	Value	Percentage (*N*)
Eosinophil count per HPF	Median: 86 (IQR: 47–131)	
Eosinophil count per mm^2^	Median: 188 (IQR: 118–300)	
Sample adequacy (basal lamina)		
Minimal (only axes)		55.1% (*n* = 43)
Scarce (<10%)		26.9% (*n* = 21)
Adequate (>10%)		15.4% (*n* = 12)
Eosinophil degranulation		89.7% (*n* = 70)
Basal lamina hyperplasia		71.8% (*n* = 56)
Eosinophil abscesses		50% (*n* = 39)
Intersticial IgG4 + staining		23.1 (*n* = 39)
Total plasma cell count	Median 80 (IQR: 30–180)	
At least one IgG4 + plasma cell per HPF		64.5 (*n* = 49)
IgG4 + plasma cells per HPF	Median 5 (IQR: 0–23)	
>10 IgG4 + plasma cells per HPF		39.7% (*n* = 31)

HPF, high-power field; IQR, interquartile range.

### Allergy history and sensitization profiles

Family and personal histories of allergic symptoms were analyzed. Atopic dermatitis was present in 16.7% (*n* = 13), rhinoconjunctivitis in 76.9% (*n* = 60), allergic asthma in 34.6% (*n* = 27), and food allergy in 43.6% (*n* = 34). A first-degree family history of atopy was reported in 38.5% (*n* = 30).

Overall sensitization to aeroallergens was present in 80.8% (*n* = 63) of patients, with 71.8% (*n* = 56) sensitized to pollen, 46.2% (*n* = 36) to epithelia, 29.5% (*n* = 23) to mites, and 11.5% (*n* = 9) to fungi. Sensitization to foods was observed in 62.8% (*n* = 49), with positive skin prick tests for milk in 6.4% (*n* = 5), wheat in 12.8% (*n* = 10), egg in 9% (*n* = 7), soy in 6.4% (*n* = 5), peanut in 17.9% (*n* = 14), other nuts in 35.9% (*n* = 28), fish in 3.8% (*n* = 3), seafood in 12.8% (*n* = 10), mustard in 7.7% (*n* = 6), peach in 26.9% (*n* = 21), other cereals in 17.9% (*n* = 14), and other fruits in 26.9% (*n* = 21).

Mean and median values for specific IgE to milk, wheat, egg, soy, peanut, fish, and seafood, as well as the percentage of positive values based on reference thresholds (total IgE >120 kU/L, specific IgE >0.35 kU_A_/L), are shown in Table 3.

**Table 3:** Mean and median values of serum total IgE, specific IgE, and specific IgG4 for the evaluated foods.

Inmunoglobulin	Mean ± SD	Median (IQR)	% positive*
Total IgE	228 ± KU_A_/L ± 329	130 KU_A_/L IQR (236–55)	52.6%
IgE milk	0.43 KU_A_/L ± 1.02	0.09 KU_A_/L IQR (0.18–0.04)	19.2%
IgE wheat	1.29 KU_A_/L ± 5.4	0.15 KU_A_/L IQR (0.68–0.02)	34.6%
IgE egg	0.28 KU_A_/L ± 1.15	0.06 KU_A_/L IQR (0.16–0.01)	10.3%
IgE soy	0.27 KU_A_/L ± 0.66	0.05 KU_A_/L IQR (0.23–0.01)	16.7%
IgE peanut	1.12 KU_A_/L ± 3.7	0.09 KU_A_/L IQR (0.85–0.02)	34.6%
IgE fish	0.048 KU_A_/L ± 0.16	0.0 KU_A_/L IQR (0.02–0.0)	6.4%
IgE seafood	0.37 KU_A_/L ± 1.64	0.03 KU_A_/L IQR (0.22–0.01)	17.9%
IgG4 milk	105 mg_A_/L ± 133.6	19 mg_A_/L IQR (11–300)	89.5%
IgG4 casein	24 mg_A_/L ± 71.3	3.5 mg_A_/L IQR (10.4–0.66)	25.8%
IgG4 β-lactoglobulin	11 mg_A_/L ± 37.6	3 mg_A_/L IQR (12.9–0.41)	30.8%
IgG4 wheat	11.5 mg_A_/L ± 47	2.2 mg_A_/L IQR (5.6–0.94)	14.3%
IgG4 egg	34.42 mg_A_/L ± 84.85	7.7 mg_A_/L IQR (14.4–2.9)	39%
IgG4 soy	0.53 mg_A_/L ± 1.08	0.20 mg_A_/L IQR (0.47–0.07)	0%
IgG4 peanut	0.90 mg_A_/L ± 1.23	0.43 mg_A_/L IQR (1.2–0.10)	0%
IgG4 fish	2.0 mg_A_/L ± 12.9	0.04 mg_A_/L IQR (0.32–0.01)	2.6%
IgG4 seafood	0.07 mg_A_/L ± 0.10	0.04 mg_A_/L IQR (0.08–0.02)	0%

*Total IgE >120 KU/L, specific IgE >0.35 KU_A_/L. No universally established positivity threshold exists for serum food-specific IgG4. A cutoff of 10 mg_A__/_L was adopted based on Lim et al. (19).

The mean and median values of specific IgG4 for casein and β-lactoglobulin (milk), wheat, egg, soy, peanut, fish, and seafood are presented in Table 3.

The median pre-treatment peripheral blood eosinophilia was 460 cells/µL (IQR: 290–636), whereas post-treatment it was 210 cells/µL (IQR: 170–430).

### Associations with poor outcomes and Serum IgG4 Levels

Patients with poor overall outcomes were defined as those who did not achieve a histological response and/or required second-line therapy. These patients exhibited a higher proportion of eosinophil abscesses in esophageal biopsies (60%) compared to those with better outcomes (36.7%) (*p* = 0.04; RR = 1.77; 95% CI: 1–3.1) and a higher eosinophil count per HPF at diagnosis (112 eos/HPF vs. 78 eos/HPF) (*p* = 0.003; r = 31). In patients who did not show a histological response to first-line treatment, a significantly higher concentration of specific serum IgG4 was observed for the following food groups: casein (31.8 mg_A_/L vs. 18.6 mg_A_/L; *p* = 0.03; r = 0.26), egg (45.46 mg_A_/L vs. 19.2 mg_A_/L, *p* = 0.03; r = 0.27), soy (0.69 KU_A_/L vs. 0.22 KU_A_/L; *p* = 0.04; r = 0.23) and peanut (0.95 KU_A_/L vs. 0.64 KU_A_/L; *p* = 0.03; r = 0.24).

### Comparison of patients with positive and negative IgG4 staining in esophageal biopsies

A comparative analysis of patients with positive and negative IgG4 histological staining was performed, evaluating clinical, endoscopic, histological, allergic, and outcome characteristics, summarized in Table 4.

**Table 4:** Comparative analysis of variables related to interstitial IgG4 staining (Chi-square tests).

Characteristics	Definition	Positive histological staining %(n)	Negative histological staining %(n)	*p*-value
Male Gender		80.6 (25)	70.2 (33)	0.30
Symptoms				
	Dysphagia	87.1 (27)	78.7 (37)	0.34
	Food impaction	51.6 (16)	42.6 (20)	0.43
	Heartburn	32.3 (10)	21.3 (10)	0.27
	Chest pain	0	6.4 (3)	0.15
Endoscopy				
(EFERS)	Normal	9.7 (3)	6.4 (3)	0.56
	Edema	16.1 (5)	2.1 (1)	**0**.**03**
	Rings	67.7 (21)	48.9 (23)	0.10
	Exudates	38.7 (12)	46.8 (22)	0.48
	Furrows	83.9 (26)	85.1 (40)	0.88
	Stricture	6.5 (2)	4.3 (2)	0.66
Outcomes				
	PPI responders	51.9 (14)	60 (27)	0.49
	Histological response	34.6 (9)	36.1 (13)	0.9
	Need for second-line treatment	64.5 (19)	41.3 (20)	**0**.**04**
Allergy				
	Atopic dermatitis	12.9 (9)	19.1 (4)	0.46
	Rhinoconjunctivitis	80.6 (25)	74.5 (35)	0.52
	Allergic asthma	32.3 (10)	36.2 (17)	0.72
	Food allergy	51.6 (16)	38.3 (18)	0.24
	Family history of atopy	43.3 (13)	36.2 (17)	0.60
	Aeroallergen sensitization (prick tests)	80 (24)	83 (39)	0.74
	Pollen	76.7 (23)	70.2 (33)	0.53
	Epithelia	46.7 (14)	46.8 (22)	0.99
	Mites	10 (3)	42.6 (20)	**0**.**02**
	Fungi	6.7 (2)	14.9 (7)	0.27
	Food sensitization (prick tests)	73.3 (22)	57.4 (27)	0.15
	Milk	6.7 (2)	6.4 (3)	0.96
	Wheat	3.3 (1)	19.1 (9)	**0**.**04**
	Egg	13.3 (4)	6.4 (3)	0.30
	Soy	0	10.6 (5)	0.078
	Peanut	10 (3)	23.4 (11)	0.13
	Nuts	36.7 (17)	36.2 (11)	0.96
	Fish	0	6.4 (3)	0.15
	Seafood	14.9 (7)	10 (3)	0.53

**Figure 2:**
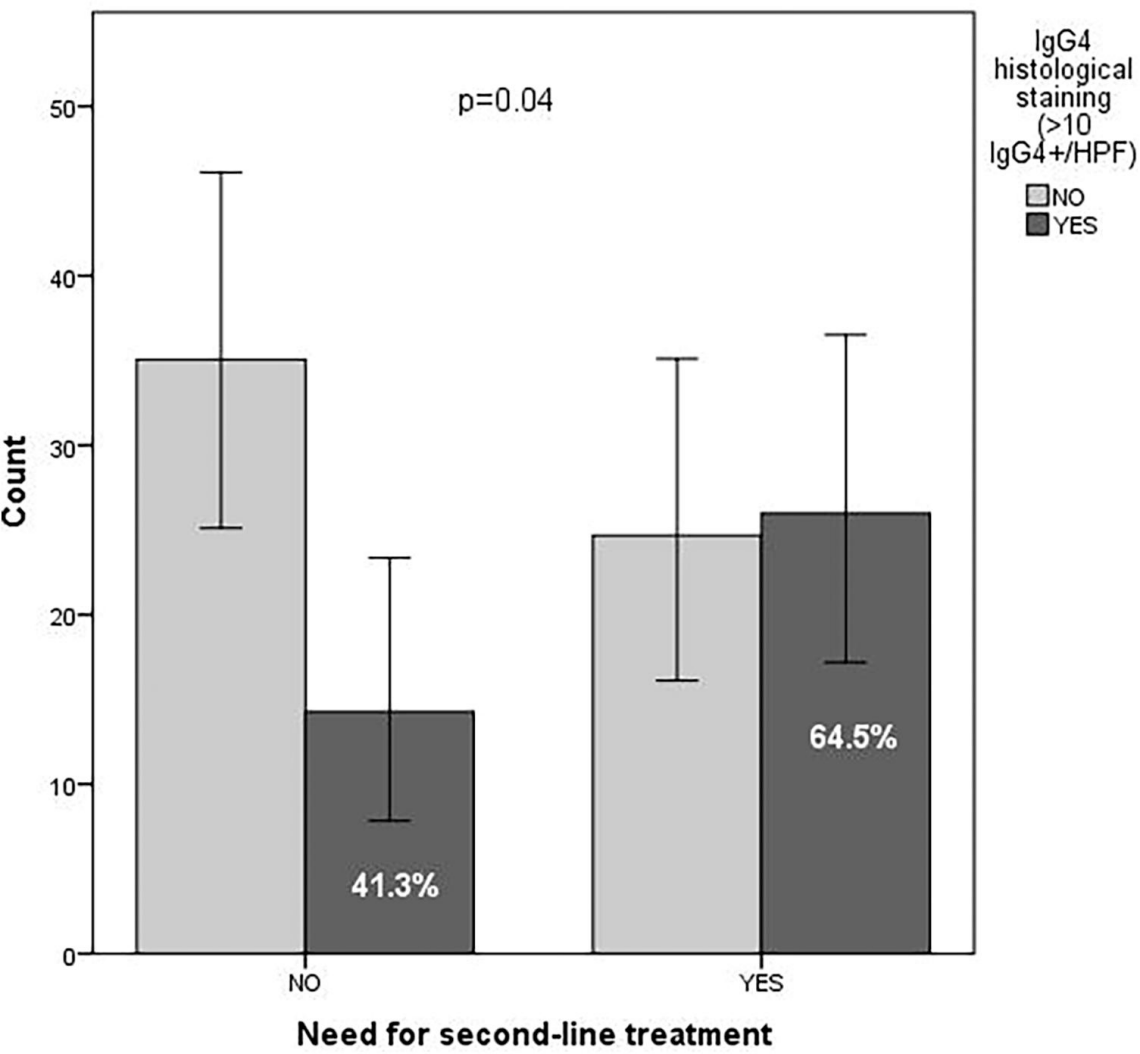
Patient counts stratified by need for second-line treatment and IgG4 histological staining status (>10 IgG4+/HPF). Light bars represent negative staining (NO); dark bars represent positive staining (YES). Patients with positive staining have greater need for second-line treatment (64.5% vs. 41.3%). Chi-square test, *p* = 0.04; RR = 2.5 (95% CI: 1–6.6).

Patients with positive histological staining exhibited a significantly higher proportion of endoscopic edema (16.1%) compared to those with negative staining (2.1%) (*p* = 0.045; RR = 8.8; 95% CI: 1–79.8) and a greater need for second-line treatment (64.5% vs. 41.3%) (*p* = 0.04; RR = 2.5; 95% CI: 1–6.6) (Figure 2).

The comparative analysis of total serum IgE, specific IgE, and specific IgG4 concentrations, along with interstitial IgG4 staining, is summarized in Table 5.

**Table 5:** Relationship between total serum IgE, specific IgE, and specific IgG4 concentrations with interstitial IgG4 staining (Mann–Whitney *U*-tests).

Inmunoglobulin	Positive histological staining median (IQR)	Negative histological staining—median (IQR)	*P*-value
IgE	120 KU_A_/L (146.5)	132 KU_A_/L (169.3)	0.93
IgE milk	0.1 KU_A_/L (0.13)	0.09 KU_A_/L (0.14)	0.56
IgG4 casein	7.3 mg_A_/L (25.15)	2.07 mg_A_/L (4.78)	**0**.**005**
IgG4 β-lactoglobulin	3.8 mg_A_/L (15.2)	2.39 mg_A_/L (8.76)	0.07
IgE wheat	0.15 KU_A_/L (0.47)	0.22 KU_A_/L (0.58)	0.76
IgG4 wheat	4.62 mg_A_/L (8.57)	1.97 mg_A_/L (3.69)	0.84
IgE egg	0.08 KU_A_/L (0.13	0.04 KU_A_/L (0.14)	0.13
IgG4 egg	12.7 mg_A_/L (14.5)	5.82 mg_A_/L (9.85)	**0**.**01**
IgE soy	0.13 KU_A_/L (0.21)	0.05 KU_A_/L (0.2)	0.73
IgG4 soy	0.32 mg_A_/L (0.68)	0.18 mg_A_/L (0.41)	0,12
IgE peanut	0.1 KU_A_/L (0.83)	0.08 KU_A_/L (0.64)	0.96
IgG4 peanut	0.91 mg_A_/L (1.29)	0.27 mg_A_/L (0.85)	0.06
IgE fish	0 KU_A_/L (0.12)	0 KU_A_/L (0.01)	0.14
IgG4 fish	0.21 mg_A_/L (1.23)	0.04 mg_A_/L (0.15)	0.88
IgE seafood	0.03 KU_A_/L (0.27)	0.03 KU_A_/L (0.21)	0.78
IgG4 seafood	0.05 mg_A_/L (0.18)	0.03 mg_A_/L (0.06)	**0**.**03**

Values in bold are statistically significant.

**Figure 3:**
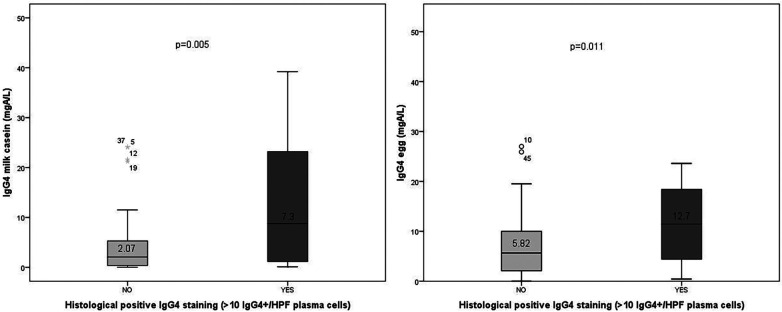
Serum-specific IgG4 levels (mgA/L) to casein and egg in patients with positive (*n* = 31) vs. negative (*n* = 47) IgG4 histological staining. Mann–Whitney *U*-test; casein: *p* = 0.005, r = 0.31; egg: *p* = 0.011, r = 0.28.

Patients with positive IgG4 histological staining exhibited significantly higher mean concentrations of serum IgG4 specific to milk casein (50.1 mg_A_/L; SD = 45.1) compared to those with negative staining (11.2 mg_A_/L; SD = 102.5) (r = 0.31; *p* = 0.005). They also showed higher mean concentrations of IgG4 specific to egg (63.4 mg_A_/L; SD = 114.2) compared to negatives (20.1 mg_A_/L; SD = 62.3) (r = 0.28; *p* = 0.011) and seafood (0.08 mg_A_/L; SD = 0,09) compared to negatives (0.05 mg_A_/L; SD = 0,10) (r = 0.23; *p* = 0.037) (Figure 3).

In the multivariate analysis using binary logistic regression, including variables with a significance level <0.1 (IgG4 casein, IgG4 lactoglobulin, IgG4 egg, IgG4 shellfish, need for second treatment, edema on endoscopy, and positive prick test to wheat, soy, and mites), only casein-specific IgG4 retained statistical significance (*p* = 0.04; OR = 1.08, 95% CI: 1–1.17).

## Discussion

In this single-centre exploratory study, esophageal tissue IgG4 staining and serum food-specific IgG4, particularly against casein and egg, were associated with more severe disease features and a higher likelihood of requiring second-line treatment in adult patients with eosinophilic esophagitis. Thus, we found that almost 40% of patients showed positive IgG4 staining, which was associated with a worse clinical course. Additionally, serum sIgG4 levels to milk and egg were associated with infiltration of IgG4-positive plasma cells in esophageal tissue and were also associated with a worse disease prognosis.

Recent studies suggest that IgG4 is involved in the pathogenesis of EoE. Thus, Rosenberg et al. (18) conducted a study involving 17 pediatric EoE patients and 19 controls, measuring levels of IgA, IgE, IgM, IgG, and IgG subclasses. The authors concluded that IgG subclasses, IgA, and IgM (but not IgE) were elevated in EoE subjects, with IgG4 showing the most significant difference, with levels up to 21 times higher in EoE subjects than controls.

Notwithstanding, there is considerable controversy in the literature regarding the utility of tissue immunostaining for IgG4 in esophageal biopsies to diagnose EoE in routine clinical practice. The study by Pope et al., involving 39 patients (31 with EoE and 8 controls), reported a sensitivity of only 48% with 100% specificity. No correlation was found between tissue IgG4 and peak eosinophil counts, nor was there a relationship between the density of IgG4-positive plasma cells and lamina propria fibrosis, a hallmark of EoE (10). On the contrary, Mohammad et al. (11) published a study with 41 pediatric patients and 10 controls, identifying a strong association between the density of IgG4-positive plasma cells in the esophageal lamina propria and EoE, which was more pronounced in patients with concurrent food allergies. The mean number of IgG4-positive plasma cells was 39 per high-power field in patients with active EoE (with 73% positivity) compared to none in non-EoE patients. Other authors, such as Zukerberg et al., reported 76% positivity for interstitial IgG4 staining (19). In a recent study by Lim et al. (9), positive IgG4 histological staining was observed in 13 out of 14 patients. In our study, we observed positivity (>10 IgG4-positive plasma cells) in approximately 40% of cases, which is lower than the rate reported in other series. When considering IgG4-positive plasma cells, the percentage rose to 65% of patients, a finding more consistent with published data. Additionally, we noted that patients with positive IgG4 histological staining were associated with endoscopic findings such as edema and exhibited more severe disease progression, requiring greater second-line treatment. Furthermore, patients with poorer outcomes showed a higher proportion of serum IgG4 specific to certain foods (mainly casein and egg).

Weidlich et al. (20) prospectively collected data from 17 patients with EoE, who were evaluated before and after budesonide treatment, and compared them with 14 non-EoE reflux patients. Total serum IgG4 levels in EoE patients were significantly higher than in those diagnosed with gastroesophageal reflux disease (GERD). Additionally, the number of IgG4-positive plasma cells was reduced post-treatment. No IgG4 histological staining was detected in GERD patients. Few studies have investigated the role of food-specific IgG4 and its relationship with EoE. In 2023, Masuda et al. (21) published a study evaluating food-specific IgG4 in blood and biopsies from the esophagus, stomach, and duodenum in 15 healthy controls, 24 patients with active EoE, and 8 patients with EoE in remission. The results demonstrated that levels of IgG4 specific to milk and wheat were elevated in plasma and the upper gastrointestinal tract (esophagus and duodenum) of EoE patients compared to controls. These elevated IgG4 levels correlated with endoscopic findings (such as inflammation and stenosis) and the severity of eosinophilic infiltration in the esophageal mucosa. Possible mechanisms could include the formation of IgG4-antigen immune complexes depositing in esophageal tissue; IgG4-antigen complexes binding to FGFR receptors on immune cells, such as eosinophils or mast cells; or inducing an aberrant allergen response (22, 23).

The role of food-specific IgG4 in identifying food triggers remains unresolved. Lim et al. (9) published a prospective study with 22 patients with active EoE and 13 controls. They analyzed serum IgG4 specific to milk, wheat, egg, soy, peanut, and seafood, followed by a targeted elimination diet for foods with IgG4 levels >10 mg_A_/L (considered positive). They observed improvement in dysphagia symptoms in all patients and a histological response in 45% of patients after 6 weeks of diet. Following the diet, specific IgG4 levels did not decrease despite the histological response. Among the foods studied, milk was positive (milk-specific IgG4) in 19 out of 22 patients (86.4%), followed by wheat (13/22, 59.1%).

In our study, we also observed an increase in food-specific IgG4 in patients with positive IgG4 histological staining. Among the foods involved (casein, egg, and seafood), casein exhibited the strongest statistical association, supporting the findings of Masuda et al. (21) and Lim et al. (9), which suggest that milk may be one of the primary foods implicated in EoE pathogenesis.

A significant issue is that serum food-specific IgG4 threshold levels are not standardized. Lim et al. (9) proposed a positivity threshold of 10 mg_A_/L. With this criterion, we observed a very high milk positivity rate, similar to that reported by Lim et al. (9). However, when evaluating casein and β-lactoglobulin separately, this percentage decreased. For seafood, soy, and peanut, although a statistical association was observed, the mean and median values were below 10 mg_A_/L, which would be interpreted as negative.

Although in our study, sample adequacy based on basal lamina characteristics was minimal (only axes) in 55.1% (*n* = 43), scarce (<10%) in 26.9% (*n* = 21), and adequate (>10%) in 15.4% (*n* = 12) of patients, these findings are consistent with those published, in which only around 50% of esophageal biopsies contained evaluable lamina propria, often due to small sample size, superficial biopsy depth, and processing artifacts. The unique anatomical structure of the esophagus (multilayered, with the lamina propria forming periodic papillary protrusions) often results in absent or inadequate lamina propria in samples, as noted in the European consensus on EoE, which excludes lamina propria fibrosis from standard histologic scoring due to its frequent underrepresentation (12, 24).

This study is not without limitations. It is a unicentric study; however, this could confer greater uniformity to data. In addition, threshold levels for specific IgG4 levels are not standardized, although a suggested limit (9) was used in our study. Additionally, data related to allergens other than milk should be considered with caution, as levels were low and did not reach statistical significance in multiple comparisons.

In conclusion, IgG4 appears to be involved in the pathogenesis of EoE, as shown by positive staining in esophageal biopsies and the detection of food-specific IgG4 in serum. Furthermore, we observed that serum IgG4 directed against casein and, to a lesser extent, egg, was associated with infiltration of IgG4-positive plasma cells in esophageal tissue and a worse disease prognosis. That fact could help identify food triggers in EoE patients and guide personalized dietary interventions, though the influence of dietary habits should be considered. These results should be further evaluated to determine whether they would qualify for a diagnostic or therapeutic biomarker.

## Data Availability

The raw data supporting the conclusions of this article will be made available by the authors, without undue reservation.

## References

[1] Dellon ES Liacouras CA Molina-Infante J Furuta GT Spergel JM Zevit N Updated international consensus diagnostic criteria for eosinophilic esophagitis: proceedings of the AGREE conference. Gastroenterology. (2018) 155(4):1022–33. 10.1053/j.gastro.2018.07.009 30009819 PMC6174113

[2] De Rooij WE Barendsen ME Warners MJ van Rhijn AO Verheij WJ Bruggink AH Emerging incidence trends of eosinophilic esophagitis over 25 years: results of a nationwide register-based pathology cohort. Neurogastroenterol Motil. (2021) 33(7):e14072. 10.1111/nmo.14072 33426755 PMC8365671

[3] Lucendo AJ Arias-Gonzalez L Molina-Infante J Arias Á. Systematic review: health-related quality of life in children and adults with eosinophilic oesophagitis-instruments for measurement and determinant factors. Aliment Pharmacol Ther. (2017) 46:401–9. 10.1111/apt.14194 28639700

[4] Lim AH Wong S Nguyen NQ. Eosinophilic esophagitis and IgG4: is there a relationship? Dig Dis Sci. (2021) 66(12):4099–108. 10.1007/s10620-020-06788-0 33534011

[5] Clayton F Fang JC Gleich GJ Lucendo AJ Olalla JM Vinson LA Eosinophilic esophagitis in adults is associated with IgG4 and not mediated by IgE. Gastroenterology. (2014) 147(3):602–9. 10.1053/j.gastro.2014.05.036 24907494

[6] Noti M Wojno ED Kim BS Siracusa MC Giacomin PR Nair MG Thymic stromal lymphopoietinelicited basophil responses promote eosinophilic esophagitis. Nat Med. (2013) 19(8):1005–13. 10.1038/nm.3281 23872715 PMC3951204

[7] Wong S Smith G Ruszkiewicz A Nguyen NQ. Distinguishing gastroesophageal reflux disease and eosinophilic esophagitis in adults: the role of esophageal mucosal immunoglobulin G4. JGH Open. (2020) 4(5):851–5. 10.1002/jgh3.12327 33102754 PMC7578275

[8] Wright BL Kulis M Guo R Orgel KA Wolf WA Burks AW Food-specific IgG4 is associated with eosinophilic esophagitis. J Allergy Clin Immunol. (2016) 138(4):1190–2. 10.1016/j.jaci.2016.02.024 27130859 PMC5053831

[9] Lim AHW Ngoi B Perkins GB Wong S Whitelock G Hurtado P Outcomes of serum food-specific immunoglobulin G4 to guide elimination diet in patients with eosinophilic esophagitis. Am J Gastroenterol. (2024) 119:1066–73. 10.14309/ajg.0000000000002678 38299582

[10] Pope AE Stanzione N Naini BV Garcia-Lloret M Ghassemi KA Marcus EA Esophageal IgG4: clinical, endoscopic, and histologic correlations in eosinophilic esophagitis. J Pediatr Gastroenterol Nutr. (2019) 68(5):689–94. 10.1097/MPG.0000000000002227 30540707 PMC8384046

[11] Mohammad N Avinashi V Chan E Vallance BA Portales-Casamar E Bush JW. Pediatric eosinophilic esophagitis is associated with increased Lamina Propria immunoglobulin G4-positive plasma cells. J Pediatr Gastroenterol Nutr. (2018) 67(2):204–9. 10.1097/MPG.0000000000001949 29509633

[12] Lucendo AJ Molina-Infante J Arias Á von Arnim U Bredenoord AJ Bussmann C Guidelines on eosinophilic esophagitis: evidence-based statements and recommendations for diagnosis and management in children and adults. United European Gastroenterol J. (2017) 5(3):335–58. 10.1177/2050640616689525 28507746 PMC5415218

[13] Deshpande V Zen Y Chan JK Yi EE Sato Y Yoshino T Consensus statement on the pathology of IgG4-related disease. Mod Pathol. (2012) 25(9):1181–92. 10.1038/modpathol.2012.72 22596100

[14] Stone JH Zen Y Deshpande V. IgG4-related disease. N Engl J Med. (2012) 366(6):539–51. 10.1056/NEJMra1104650 22316447

[15] Klimek L Hoffmann HJ Kalpaklioglu AF Demoly P Agache I Popov TA In-vivo diagnostic test allergens in Europe: A call to action and proposal for recovery plan-An EAACI position paper. Allergy. (2020) 75(9):2161–9. 10.1111/all.14329 32306414

[16] Macías EM Velasco Guardado A Dávila I. Measuring food-specific-IgG4s with milk. Am J Gastroenterol. (2025) 120(12):2965–66. 10.14309/ajg.0000000000003474 40323028

[17] Bel Imam M Iwasaki S Lems S Cevhertas L Westermann P Larsen LB Circulating food allergen-specific antibodies, beyond IgG4, are elevated in eosinophilic esophagitis. Clin Exp Allergy. (2025) 55(10):916–27. 10.1111/cea.70055 40230181 PMC12515539

[18] Rosenberg CE Mingler MK Caldwell JM Collins MH Fulkerson PC Morris DW Esophageal IgG4 levels correlate with histopathologic and transcriptomic features in eosinophilic esophagitis. Allergy. (2018) 73(9):1892–901. 10.1111/all.13486 29790577 PMC6119084

[19] Zukerberg L Mahadevan K Selig M Deshpande V. Oesophageal intrasquamous IgG4 deposits: an adjunctive marker to distinguish eosinophilic oesophagitis from reflux oesophagitis. Histopathology. (2016) 68(7):968–76. 10.1111/his.12892 26466342 PMC5717757

[20] Weidlich S Nennstiel S Jesinghaus M Brockow K Slotta-Huspenina J Bajbouj M Igg4 is elevated in eosinophilic esophagitis but not in gastroesophageal reflux disease patients. J Clin Gastroenterol. (2020) 54(1):43–9. 10.1097/MCG.0000000000001154 30614939

[21] Masuda MY LeSuer WE Horsley-Silva JL Putikova A Buras MR Gibson JB Food-specific IgG4 is elevated throughout the upper gastrointestinal tract in eosinophilic esophagitis. Dig Dis Sci. (2023) 68(6):2406–13. 10.1007/s10620-023-07924-2 36973521 PMC10198037

[22] Piseddu I Mayerle J Mahajan UM Boehmer DFR. The dual role of IgG4 in immunity: bridging pathophysiology and therapeutic applications. Gut. (2025) 74(9):1528–38. 10.1136/gutjnl-2025-335375 40592566 PMC12418560

[23] Li Z Yan S Zhang J Li H Wu Z Cheng L Allergen-specific IgE and IgG4 signatures in IgG4-related disease revealed by a large-scale PhIP-seq study. Allergy. (2025):1–15. 10.1111/all.70162 41267614

[24] Pouw RE Barret M Biermann K Bisschops R Czakó L Gecse KB Endoscopic tissue sampling - part 1: upper gastrointestinal and hepatopancreatobiliary tracts. European Society of Gastrointestinal Endoscopy (ESGE) Guideline. Endoscopy. (2021) 53(11):1174–88. 10.1055/a-1611-5091 34535035

